# The Use of Self-Sampling Devices via a Smartphone Application to Encourage Participation in Cervical Cancer Screening: A Pilot Study

**DOI:** 10.3390/jcm14155569

**Published:** 2025-08-07

**Authors:** Francesco Plotti, Fernando Ficarola, Giuseppina Fais, Carlo De Cicco Nardone, Roberto Montera, Daniela Luvero, Gianna Barbara Cundari, Alice Avian, Elisabetta Riva, Santina Castriciano, Silvia Angeletti, Massimo Ciccozzi, Roberto Angioli, Corrado Terranova

**Affiliations:** 1 Research Unit of Gynaecology, Fondazione Policlinico Universitario Campus Bio-Medico, Via Alvaro del Portillo 200, 00128 Rome, Italy; f.plotti@policlinicocampus.it (F.P.); c.decicconardone@policlinicocampus.it (C.D.C.N.); r.montera@policlinicocampus.it (R.M.); d.luvero@policlinicocampus.it (D.L.); g.cundari@policlinicocampus.it (G.B.C.); r.angioli@policlinicocampus.it (R.A.); c.terranova@policlinicocampus.it (C.T.); 2 Research Unit of Gynaecology, Department of Medicine and Surgery, Università Campus Bio-Medico Di Roma, Via Alvaro del Portillo 200, 00128 Rome, Italy; 3 Obstetrics and Gynecological Unit, Department of Woman’s and Child’s Health, San Camillo-Forlanini Hospital, 00152 Rome, Italy; 4 Division of Gynecology and Obstetrics, Department of Surgical Sciences, University of Cagliari, 09124 Cagliari, Italy; g.fais91@gmail.com; 5 Molecular Genetics and Biotechnology PhD Study Program, University of Nova Gorica, 5000 Nova Gorica, Slovenia; a.avian@ulissebiomed.com; 6 Unit of Clinical Laboratory Science, Fondazione Policlinico Universitario Campus Bio-Medico, Via Alvaro del Portillo 200, 00128 Rome, Italy; e.riva@policlinicocampus.it (E.R.); s.angeletti@policlinicocampus.it (S.A.); 7 Copan Italia Spa, 25125 Brescia, Italy; santina.castriciano@copangroup.com; 8 Research Unit in Medical Statistics and Molecular Epidemiology, Fondazione Policlinico Universitario Campus Bio-Medico, Via Alvaro del Portillo 200, 00128 Rome, Italy; m.ciccozzi@policlinicocampus.it

**Keywords:** HPV, human papillomavirus, self-sampling, screening, cervical cancer, self-screening

## Abstract

**Background:** Cervical cancer ranks among the most prevalent tumors in low-income countries, with the Pap test as one of the primary screening tools. The Pap smear detects abnormal cells, the CLART test identifies specific HPV genotypes, and HPV self-sampling allows for self-collected HPV testing. This study aimed to evaluate the feasibility of the first smartphone-based health device for home-collection HPV testing. **Methods:** Enrolled patients during the gynecological examination underwent three different samplings: Pap smear, HPV DNA genotyping test CLART, and vaginal HPV-Selfy swab. Each patient received a kit including an activation code, vaginal swab, and instructions. After performing the self-sample, patients returned the kit to our laboratory. Both the samples collected by the gynecologist and those collected by the patients themselves were analyzed. **Results:** A total of 277 patients were enrolled, with 226 self-collected swabs received for analysis. The assay yielded valid results for both self-collected and clinician-collected swabs in 190 patients. When comparing these results with paired clinician-taken vaginal swabs, we observed an agreement of 95.2% (Cohen’s Kappa: 0.845). We report an agreement of 93.7% (Cohen’s Kappa: 0.798). **Conclusions:** The study demonstrated the feasibility of HPV-Selfy as a complementary tool in cervical cancer screening, especially where adherence to traditional surveillance is low.

## 1. Introduction

Cervical cancer ranks as the fourth most common neoplasm and the fourth leading cause of cancer-related mortality among women worldwide, with approximately 604,127 new cases and 341,831 deaths reported in 2020 [1]. The distribution of cervical cancer varies widely, with over 85% of the global burden occurring in low- and middle-income countries [2].

The role of human papillomavirus (HPV) as a causative agent in cervical precancerous lesions and cancer has been firmly established both epidemiologically and biologically. While HPV infection is essential, it is not sufficient for the development of cancer; other factors such as multiparity, host immune response, hormonal influences, and cigarette smoking also contribute significantly [3]. Based on these findings, the World Health Organization (WHO) recommends implementing either of the following strategies for cervical cancer prevention among the general population of women:

- HPV DNA detection in a screen-and-treat approach, commencing at the age of 30 years, with regular screening every 5 to 10 years.
- HPV DNA detection in a screen, triage, and treat approach, also starting at the age of 30 years, with regular screening intervals of 5 to 10 years.

Additionally, the WHO suggests HPV self-sampling as an adjunct screening method. Conventional cervical cancer screening methods, such as Pap smear and clinician-collected HPV testing, are highly effective but often limited by barriers such as patient discomfort, logistical challenges, and dependency on healthcare infrastructure. In contrast, self-sampling approaches, particularly those integrated with mobile health (mHealth) technologies, provide a private, convenient alternative and have demonstrated potential to improve screening coverage, especially in under-screened populations [4,5].

Self-sampling for HPV DNA has proven effective in detecting cervical cancer and is generally well-received by end-users. Indeed, most studies have demonstrated that self-sampling is highly acceptable for HPV testing, irrespective of study location, sampling method, device, setting, or participant demographics. In the majority of cases, women preferred self-sampling for HPV DNA testing over clinician sampling [3,6,7,8,9,10,11,12,13,14,15,16,17,18,19]. When given the choice between self-sampling at home or in a clinic, most women expressed a preference for home collection. However, a common limitation in home-collection acceptance is women’s lack of confidence in the accuracy of self-sampled specimens, reported in studies conducted in both high- and low-income settings [20,21]. Women generally exhibited greater confidence in clinicians’ ability to collect specimens accurately. Providing clear instructions or visual aids could help reassure women about performing self-collection at home [22]. Mobile health (mHealth) interventions could play a pivotal role in addressing these concerns. Smartphones, as widely available portable devices, are used globally by over 6.2 billion people as of 2021 [23].

Smartphone-based applications may support patients in self-collection and follow-up, particularly in remote rural areas and developing regions. In the context of HPV self-sampling, a smartphone app could offer additional support to women throughout the sample collection process, as well as in result delivery and subsequent follow-up. While mobile apps have been developed to raise awareness about HPV infection or promote HPV vaccine uptake, no app currently supports primary screening [24,25,26]. This study aimed to evaluate the feasibility of the first smartphone-based mHealth device for home-collection HPV testing.

## 2. Materials and Methods

### 2.1. Study Design

This prospective observational case–control study was conducted at the Fondazione Policlinico Universitario Campus Bio-Medico of Rome. The study adhered to the principles outlined in the Declaration of Helsinki and received approval from the Institutional Review Board of University Campus Bio-Medico of Rome (protocol code n. 73/18 approved on 18 December 2018). Female patients attending the gynecology outpatient clinic, aged between 18 and 65 years, who owned a smartphone and expressed willingness to participate, were included after providing informed consent. Pregnant women, those currently diagnosed with uterine, endometrial, vaginal, vulvar, or ovarian cancers, and individuals using vaginal ovules, creams, or undergoing vaginal douching, sexual intercourse, or menstruation within three days prior to the examination, verified by checking medical records, were excluded from the study. Patients who did not return the vaginal swab collected at home through self-sampling were excluded from the statistical analysis.

Following enrollment and consent, patients underwent a gynecological visit where their medical history was collected. Subsequently, women underwent two vaginal samplings performed by the clinician: one using FLOQSwabs^®^ (by Copan Italia S.p.A, Brescia, Italy) (the same swab provided in the home-collection kit) and another using traditional cervical sampling with a brush stored later in Thin Prep PreservCyt^®^ (Hologic BV, Zaventem, Belgium) medium for concurrent Pap smear and HPV DNA testing.

During gynecological counseling, women received a comprehensive explanation of the initiative and were provided with a home-collection kit manufactured HPV Selfy^®^ (by Ulisse Bio-Med S.p.A, Basovizza, Italy). The kit included a self-collected vaginal swab (FLOQSwabs^®^ by Copan Italia S.p.A, Brescia, Italy) with instructions for use, general procedure instructions, an activation code, and username/password to access the mobile web application, as well as an envelope for sample shipment (which included pre-marked shipment envelopes for traditional mailing and a prepaid shipment letter to be deposited at the nearest TNT^®^ Drop-point). Pap test results were communicated and sent to all enrolled patients, regardless of whether they had collected the sample at home and registered the kit online.

### 2.2. Home Self-Sampling

At home, women performed self-sampling of vaginal secretions. Through the web app, they could access a step-by-step tutorial on the self-sampling procedure. Subsequently, they were required to return the samples to the hospital, following the tutorial instructions provided on the digital platform. This involved both delivering the samples to a drop-point (TNT^®^) and using a traditional mailbox. Upon receipt of the envelopes at the hospital, the time of arrival and the status of the samples were recorded. In the event of a positive HPV DNA test and/or a positive Pap test result, the patient received an email with instructions to contact the gynecologist for an appointment to receive her results and discuss the appropriate follow-up steps. Patients were also asked to provide feedback on their experience using the platform, including whether they encountered any difficulties and whether the instructions provided were sufficient to facilitate the activation of the kit and the self-collection process.

### 2.3. HPV Testing

According to the manufacturer’s instructions, both self-collected and clinician-collected vaginal samples underwent testing using the HPV Selfy^®^ assay (by Ulisse Bio-Med S.p.A, Basovizza, Italy). HPV Selfy^®^ is a CE IVD real-time full genotyping PCR-based screening test capable of detecting and genotyping 14 high-risk HPV types (16, 18, 31, 33, 35, 39, 45, 51, 52, 56, 58, 59, 66, and 68) in a single real-time PCR reaction. The test has been validated for screening purposes not only on clinician-collected samples but also on self-collected samples in accordance with international guidelines [27]. Prior to HPV Selfy^®^ testing, samples underwent pre-treatment with Ulisse Faster DNA (by Ulisse Bio-Med S.p.A, Basovizza, Italy), a reagent that allows for the omission of DNA extraction and direct loading of raw samples into the PCR reaction after a brief pre-treatment, thereby saving time and costs. The HPV Selfy^®^ test includes a human DNA amplification control (Hemoglobin subunit beta) to assess sample quality, thereby reducing the risk of false-negative results. Analysis was conducted following the manufacturer’s protocol, utilizing a QuantStudio 5 Dx Real-Time PCR machine (Thermo Fisher Scientific; Waltham, MA, USA).

Cervical specimens were analyzed using a CE-IVD test capable of genotyping high- and low-risk HPVs: CLART HPV 2 ^®^ (Genomica, Madrid, Spain) (CLART). CLART detects 14 high-risk HPV types (16, 18, 31, 33, 35, 39, 45, 51, 52, 56, 58, 59, 66, and 68) as well as 21 low-risk and probable high-risk HPV types (6, 11, 40, 42, 43, 44, 54, 61, 62, 70, 71, 72, 81, 83, 84, 85, 89, 26, 53, 73, and 82) using PCR amplification followed by a microarray hybridization assay. Cervical smear slides were Pap-stained, and histotechnicians interpreted the results following the Bethesda 2001 classification [28].

### 2.4. Statistical Analysis

Demographic and clinical data for each enrolled patient were collected using a case report form and stored in a database using Excel^®^ version 13.0 for Windows. Data analysis was conducted exclusively on patients who performed self-sampling at home and returned the samples. Among these patients, only those whose samples were deemed readable due to correct self-collection were included in the evaluation. A resulting *p*-value was calculated as an indicator of significance, with α set at 0.05. Statistical analysis was performed using MedCalc Statistical^®^ Software version 16.4.3 and GraphPad Prism^®^ version 7.00 for Windows. Cohen’s Kappa index was employed to assess agreement between different tests [29]. A minimum of 186 patients was determined as necessary to achieve statistical significance in this study, calculated based on 95% power to detect a significant difference in the two sampling methods at a 5% significance level. Accounting for a dropout risk of 33%, a total of 277 patients were enrolled to maintain the study’s statistical power.

## 3. Results

From January to July 2019, 277 patients were enrolled at Fondazione Policlinico Universitario Campus Bio-Medico of Rome, of which 226 completed the study by successfully sending back the sample (226/277 = 81.6%), whereas 51 women did not (51/277 = 18.4%) and were excluded from the final analysis. The main clinical and demographic characteristics and patient compliance are reported in Table 1.

**Figure 1:**
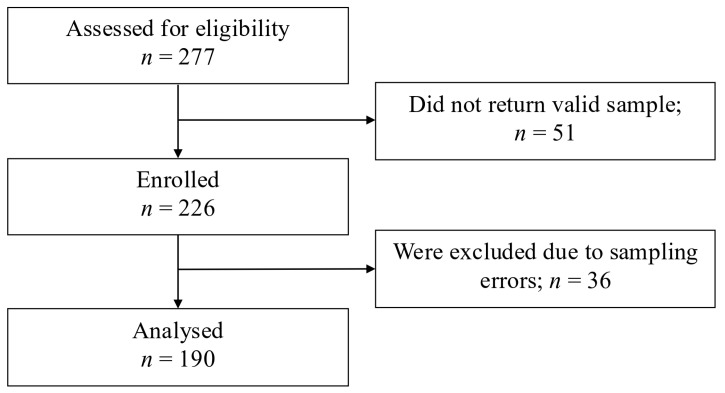
Trial profile.

The study flow diagram is reported in Figure 1.

Among the 226 women who correctly completed the procedure, 196 women (86.7%) shipped the kit back through the traditional mailbox, while 30 women (13.3%) used the drop-point courier service. The average time from kit activation to final delivery at the hospital was 2 days for the drop-point courier group, with a minimum of 1 day and a maximum of 4 days. For the traditional mailbox group, the average time was also 2 days, with a minimum of 1 day and a maximum of 13 days. Seventy-five percent of samples were received within 3 days from activation. For the study, we considered 190 paired samples that provided valid results in all four analyses performed: Pap test and CLART test on clinician-collected vaginal swabs, as well as the self-sampling tests on both clinician-collected and home-collected vaginal swabs. The results of HPV testing were analyzed for positivity to high-risk HPV strains, and corresponding results along with genotyping information of the detected strains are presented in Table 2.

When comparing the results of the self-sampling test performed on self-collected swabs with the same assay conducted on paired clinician-taken vaginal swabs, we observed an agreement of 95.3% (Cohen’s Kappa: 0.845). This indicates that neither shipping method significantly affects the test results. Furthermore, when comparing the self-collection method with the CE-IVD test (CLART) performed on the same clinician-collected vaginal specimen, we found substantial agreement between the two methods, with a total agreement of 93.7% and Cohen’s Kappa coefficient of 0.798 (see Table 3).

Overall, we identified 27 cases of HPV coinfections (double infections) and 39 single infections. The frequency of each genotype is detailed in Table 2, with the most prevalent HPV type being HPV 66 (10 infections), followed by HPV 58 (9 infections). As previously mentioned, patients in this study also underwent a Pap smear test during their visit. Of the 190 samples correctly analyzed, 25 yielded abnormal results (13.1%): 15 cases of ASCUS and 10 cases of LSIL. Among the 25 patients with abnormal Pap smear results, 16 (64%) also tested positive for HPV infection: 6 ASCUS patients exhibited HPV infection (4 with high-risk HPV and 2 with low-risk HPV), while 9 LSIL patients had HPV infection (7 with high-risk HPV and 2 with low-risk HPV). Among the 165 patients with normal Pap smear results, 38 (23%) also had an HPV infection (24 with high-risk HPV and 14 with low-risk HPV). Notably, among vaccinated patients, none tested positive for the serotypes targeted by the vaccine; however, 4 out of 36 vaccinated patients (11.11%) tested positive for high-risk HPV types (HPV 31, HPV 51, HPV 56, and HPV 59) not covered by the vaccination.

Regarding the satisfaction questionnaire, 90 out of 190 patients (47.4%) completed it. Of these, 60 (66%) reported that the self-sampling test was easy to perform, 80 (88%) found it less painful than the clinician-performed test, and 61 (67%) preferred the self-sampling method over the conventional test. Additionally, patients provided feedback on the general procedure of the screening project based on self-collection, rating their satisfaction on a scale from very low (1) to very high (10). On average, women rated the project proposal highly, with an average satisfaction index of 9.01. Furthermore, most women appreciated the support provided by the smartphone web app, with 72.9% describing it as “simple” and 22.1% as “user-friendly”.

## 4. Discussion

Cervical cancer ranks as the fourth most common cancer affecting women worldwide, and despite the introduction of vaccines, screening remains the primary form of prevention [30]. The Pap smear test continues to be the most widely used screening tool [31]. While HPV testing does not detect morphological alterations, it has become an integral part of clinical guidelines for cervical cancer screening in many countries [32]. However, the effectiveness of cytological and HPV screening relies heavily on participation, and most cervical carcinomas occur in women who do not undergo regular testing or have never been screened [33,34,35]. Therefore, increasing screening coverage is essential [35,36]. Reduced screening participation often stems from factors such as discomfort or embarrassment related to gynecological examinations, low education levels, and logistical challenges [37,38]. Moreover, in developing countries, a lack of resources and trained personnel often limits cancer prevention efforts to screen-and-treat approaches [39,40,41]. The use of a self-sampling test, as utilized in this study, could facilitate the detection of high-risk HPV genotypes and improve screening coverage [42]. To address low patient confidence in using self-screening tools, we propose several strategies: integrating step-by-step video tutorials and clear visual aids in the smartphone application, enhancing the user interface to provide real-time feedback and reassurance, and establishing remote support systems (e.g., live chat or helplines) to guide users through the process. Such interventions have been shown to improve user trust and engagement in similar mHealth initiatives [39,40].

Previous studies on self-sampling methods have evaluated both the sensitivity and accuracy of samples collected by patients compared to those collected by physicians [4]. Some studies have shown that self-sampling is more effective than reminder letters for Pap tests among patients who do not participate in regular cervical screening programs [5,43]. In our study, we observed excellent agreement between patient-collected and physician-collected samples, consistent with findings from other studies [5,44]. Despite this, most tests for detecting viral DNA rely on samples collected by clinicians and are not designed for self-use [45]. Our study demonstrated very good adherence to self-collection screening, with 226 out of 277 patients (81.58%) participating, even though the patients had already undergone molecular and cytological screening in the hospital. This high participation rate may be partially attributed to the simplicity of the procedure and the support provided by the smartphone web app, as indicated by the questionnaire results. However, it is important to note that potential bias may exist, as the enrolled women were mostly aware of the importance of regular check-ups for cervical cancer prevention. Moreover, the high participation rate in this pilot study may limit generalizability to populations with lower health literacy. Compliance was particularly high among patients with higher levels of education (diploma or degree), reaching 91.46%. These findings are consistent with a study conducted by Smith et al. in 2014 in Canada, which demonstrated the importance of awareness and education levels in screening program participation [46]. Although digital support tools can enhance adherence, the implementation of smartphone-assisted self-sampling programs may involve additional costs related to app development, maintenance, and user training. Moreover, limited digital literacy or lack of access to smartphones in certain populations could pose barriers to widespread adoption. These considerations are crucial when planning population-based interventions, especially in low-resource settings. Successful implementation would require not only technical infrastructure, but also organizational coordination with existing healthcare services to ensure follow-up of positive results and integration into national screening pathways.

Importantly, in our study, all vaccinated patients tested negative for the viral genotypes included in the vaccines. However, 11.1% of vaccinated patients still had infections with other HPV genotypes not covered by the vaccine, highlighting the importance of screening participation for vaccinated women and the value of information derived from screening tests with single-HPV full genotyping capability. Consistent with the FUTURE II Study Group findings, vaccination against HPV is highly effective (98%) in patients who have never been exposed to the virus, particularly for the HPV16 and HPV18 types under study [47]. However, vaccine efficacy appears to be lower (44%) in patients with existing intraepithelial lesions (CIN) from HPV16 and HPV18 at the time of vaccination or who are already infected with these genotypes [47].

The high adherence of patients to our study may be attributed in part to the less invasive nature of the self-sampling test, which is well tolerated and highly approved by patients. Consistent with findings from Nishimura et al., our study showed that most patients prefer self-sampling over clinician-performed tests due to its reduced invasiveness, pain, and the ability to perform the test privately without anxiety or embarrassment [22,48]. However, some patients expressed a preference for tests performed by specialists due to the clinician’s expertise in sample collection. Additionally, according to Howard et al., some patients prefer clinician-performed tests for the convenience of combining the test with a gynecological visit [21].

Despite the advantages observed, the self-sampling approach also presents practical limitations that must be carefully considered before broad implementation. In particular, the success of the procedure relies on the patient’s ability to correctly follow instructions and collect an adequate sample, which may be compromised in individuals with limited digital skills, cognitive impairments, or low health literacy. Poor sample quality or unsuccessful procedures could reduce the reliability of test results, undermining the effectiveness of screening programs. For this reason, self-sampling should ideally be offered to women who are able to fully understand and correctly perform the procedure, possibly supported by clear and accessible educational materials, user-friendly interfaces, and, when needed, professional assistance. Tailoring communication strategies and providing optional in-person or remote guidance could help ensure the equity and effectiveness of self-sampling initiatives across different population groups. The integration of these approaches into organized screening programs, supported by cost-effectiveness evaluations and real-world implementation studies, will be key to ensuring their long-term sustainability and impact.

## 5. Conclusions

The results of our study, aimed at analyzing the performance of HPV Selfy compared to the CLART method, demonstrated excellent agreement between the two. This suggests that HPV Selfy can effectively complement traditional screening methods, particularly in settings where adherence to surveillance is suboptimal. Future research should address cost-effectiveness and long-term adherence in broader population settings.

## Figures and Tables

**Table 1:** The main clinical and demographic characteristics, and patient compliance in the sending back of samples and activation of the kit.

Patients	N (%)
Age (Mean ± SD)	36.89 ± 13.22
BMI	
<18.5, *n* (%)	16 (7.1)
18.5–24.9, *n* (%)	146 (64.6)
>25, *n* (%)	50 (22.1)
N.A., *n* (%)	14 (6.2)
Number of pregnancies, (Mean ± SD)	1.10 ± 1.34
Menopause, *n* (%)	20 (8.8)
Oral contraceptive, (Mean ± SD)	39 (17.3)
Smoking	
-Smoker, *n* (%)	14 (6.2)
-Non-Smoker, *n* (%)	212 (93.8)
HPV Vaccination	
-Not vaccinated	190 (84.1)
-Vaccinated	36 (15.9)
-Cervarix^®^	7 (19.4)
-Gardasil-4^®^	26 (72.2)
-Gardasil-9^®^	2 (5.6)
Education level	
-Middle school certificate, *n* (%)	23 (8.3)
-Higher education, *n* (%)	111 (40.1)
-University degree, *n* (%)	121 (43.7)
-Not indicated, *n* (%)	22 (7.9)
Sample sent back for analysis:	
-unsuccessful, *n* (%)	51 (18.4)
-successful, *n* (%)	226 (81.6)
-Traditional mailbox	196 (86.7)
-Courier company	30 (13.3)
Digital activation of Kit	
-Unsuccessful activation, *n* (%)	62 (22.4)
-Successful activation, *n* (%)	215 (77.6)
-Immediately	165 (77)
-Within 7 days	39 (18)
-Between 8 and 18 days	11 (5)

BMI, body mass index; N, number; HPV, human papillomavirus.

**Table 2:** Analysis of the three different swabs and the corresponding HPV-HR strains.

	Self-Sampling Vaginal Swab (N = 190)	Medical Sampling Vaginal Swab (N = 190)	CLART (N = 190)
HPV-HR positivity	33 (17.4)	38 (20)	36 (18.9)
HPV-negativity	157 (82.6)	152 (80)	154 (81.1)
Coinfected HPV	10	11	18
Genotype of HPV-HR			
-Type 16	6 (3.1)	8 (4.2)	5 (2.6)
-Type 18	0 (0)	1 (0.5)	0 (0)
-Type 31	8 (4.2)	10 (5.2)	7 (3.7)
-Type 33	0 (0)	0 (0)	1 (0.5)
-Type 35	0 (0)	0 (0)	0 (0)
-Type 39	5 (2.6)	4 (2.1)	1 (0.5)
-Type 45	1 (0.5)	1 (0.5)	1 (0.5)
-Type 51	4 (2.1)	5 (2.6)	5 (2.6)
-Type 52	3 (1.6)	2 (1.0)	3 (1.6)
-Type 56	3 (1.6)	3 (1.6)	1 (0.5)
-Type 58	8 (4.2)	7 (3.7)	8 (4.2)
-Type 59	2 (1.0)	2 (1.0)	2 (1.0)
-Type 66	4 (2.1)	5 (2.6)	9 (4.7)
-Type 68	2 (1.0)	3 (1.6)	0 (0)

Coinfected HPV: defined as detection of two or more high-risk HPV genotypes in the same sample; HPV, human papillomavirus; HPV-HR, high-risk human papillomavirus.

**Table 3:** Diagnostic performance of different vaginal swab methodologies compared.

	CLART ^1^ vs. HPV Selfy ^1^	CLART ^1^ vs. HPV Selfy ^2^	HPV ^2^ vs. HPV Selfy ^1^
Sensitivity	86.1% (95%CI: 70.50–95.33%)	77.8% (95%CI: 60.85–89.88%)	81.5% (95%CI: 65.67–92.26%)
Specificity	95.4% (95%CI: 90.86–98.15%)	96.8% (95%CI: 92.59–98.94%)	98.7% (95%CI: 95.33–99.84%)
Accuracy	93.7% (95%CI: 89.23–96.69%)	93.2% (95%CI: 88.58–96.31%)	95.3% (95%CI: 91.20–97.81%)
PPV	81.6% (95%CI: 67.97–90.24%)	84.8% (95%CI: 69.91–93.10%)	93.9% (95%CI: 79.51–98.41%)
NPV	96.7% (95%CI: 92.87–98.52%)	94.9% (95%CI: 90.99–97.17%)	95.5% (95%CI: 91.65–97.67%)
K Cohen	79.9% (95%CI: 68.90–90.80%)	77.0% (95%CI: 65.10–88.90%)	84.4% (95%CI: 74.60–94.30%)
OA agrement	93.7%	93.2%	95.0%

HPV, human papillomavirus; NPV, negative predictive value; OA, overall; PPV: positive predictive value. ^1^ Performed by the clinician, ^2^ self-collected at home.

## Data Availability

The raw data supporting the conclusions of this article will be made available by the authors, without undue reservation.

## References

[1] Singh D., Vignat J., Lorenzoni V., Eslahi M., Ginsburg O., Lauby-Secretan B., Arbyn M., Basu P., Bray F., Vaccarella S. (2023). Global estimates of incidence and mortality of cervical cancer in 2020: A baseline analysis of the WHO Global Cervical Cancer Elimination Initiative. Lancet Glob. Health.

[2] Ferlay J., Colombet M., Soerjomataram I., Parkin D.M., Piñeros M., Znaor A., Bray F. (2021). Cancer statistics for the year 2020: An overview. Int. J. Cancer.

[3] Bhat D. (2022). The ‘Why and How’ of Cervical Cancers and Genital HPV Infection. Cytojournal.

[4] Arbyn M., Castle P.E. (2015). Offering Self-Sampling Kits for HPV Testing to Reach Women Who Do Not Attend in the Regular Cervical Cancer Screening Program. Cancer Epidemiol. Biomark. Prev..

[5] Snijders P.J., Verhoef V.M., Arbyn M., Ogilvie G., Minozzi S., Banzi R., van Kemenade F.J., Heideman D.A., Meijer C.J. (2013). High-risk HPV testing on self-sampled versus clinician-collected specimens: A review on the clinical accuracy and impact on population attendance in cervical cancer screening. Int. J. Cancer.

[6] Modibbo F., Iregbu K.C., Okuma J., Leeman A., Kasius A., de Koning M., Quint W., Adebamowo C. (2017). Randomized trial evaluating self-sampling for HPV DNA based tests for cervical cancer screening in Nigeria. Infect. Agent Cancer.

[7] Avian A., Clemente N., Mauro E., Isidoro E., Di Napoli M., Dudine S., Del Fabro A., Morini S., Perin T., Giudici F. (2023). Clinical validation of full HR-HPV genotyping HPV Selfy assay according to the international guidelines for HPV test requirements for cervical cancer screening on clinician-collected and self-collected samples. J. Transl. Med..

[8] Mao C., Kulasingam S.L., Whitham H.K., Hawes S.E., Lin J., Kiviat N.B. (2017). Clinician and Patient Acceptability of Self-Collected Human Papillomavirus Testing for Cervical Cancer Screening. J. Womens Health (Larchmt).

[9] Ilangovan K., Kobetz E., Koru-Sengul T., Marcus E.N., Rodriguez B., Alonzo Y., Carrasquillo O. (2016). Acceptability and Feasibility of Human Papilloma Virus Self-Sampling for Cervical Cancer Screening. J. Womens Health (Larchmt).

[10] Rosenbaum A.J., Gage J.C., Alfaro K.M., Ditzian L.R., Maza M., Scarinci I.C., Felix J.C., Castle P.E., Villalta S., Miranda E. (2014). Acceptability of self-collected versus provider-collected sampling for HPV DNA testing among women in rural El Salvador. Int. J. Gynaecol. Obs..

[11] Mbatha J.N., Galappaththi-Arachchige H.N., Mtshali A., Taylor M., Ndhlovu P.D., Kjetland E.F., Baay M.F.D., Mkhize-Kwitshana Z.L. (2017). Self-sampling for human papillomavirus testing among rural young women of KwaZulu-Natal, South Africa. BMC Res. Notes.

[12] Trope L.A., Chumworathayi B., Blumenthal P.D. (2013). Feasibility of community-based careHPV for cervical cancer prevention in rural Thailand. J. Low. Genit. Tract Dis..

[13] Winer R.L., Gonzales A.A., Noonan C.J., Cherne S.L., Buchwald D.S. (2016). Collaborative to Improve Native Cancer Outcomes (CINCO). Assessing Acceptability of Self-Sampling Kits, Prevalence, and Risk Factors for Human Papillomavirus Infection in American Indian Women. J. Community Health.

[14] Reisner S.L., Deutsch M.B., Peitzmeier S.M., White Hughto J.M., Cavanaugh T.P., Pardee D.J., McLean S.A., Panther L.A., Gelman M., Mimiaga M.J. (2018). Test performance and acceptability of self- versus provider-collected swabs for high-risk HPV DNA testing in female-to-male trans masculine patients. PLoS ONE.

[15] Aiko K.Y., Yoko M., Saito O.M., Ryoko A., Yasuyo M., Mikiko A.S., Takeharu Y., Fumiki H., Etsuko M. (2017). Accuracy of self-collected human papillomavirus samples from Japanese women with abnormal cervical cytology. J. Obs. Gynaecol. Res..

[16] Hanley S.J., Fujita H., Yokoyama S., Kunisawa S., Tamakoshi A., Dong P., Kobayashi N., Watari H., Kudo M., Sakuragi N. (2016). HPV self-sampling in Japanese women: A feasibility study in a population with limited experience of tampon use. J. Med. Screen..

[17] Jones H.E., Wiegerinck M.A., Nieboer T.E., Mol B.W., Westhoff C.L. (2008). Women in the Netherlands prefer self-sampling with a novel lavaging device to clinician collection of specimens for cervical cancer screening. Sex. Transm. Dis..

[18] Ortiz A.P., Alejandro N., Pérez C.M., Otero Y., Soto-Salgado M., Palefsky J.M., Tortolero-Luna G., Romaguera J. (2012). Acceptability of cervical and anal HPV self-sampling in a sample of Hispanic women in Puerto Rico. P. R. Health Sci. J..

[19] Phoolcharoen N., Kantathavorn N., Krisorakun W., Taepisitpong C., Krongthong W., Saeloo S. (2018). Acceptability of Self-Sample Human Papillomavirus Testing Among Thai Women Visiting a Colposcopy Clinic. J. Community Health.

[20] Fargnoli V., Petignat P., Burton-Jeangros C. (2015). To what extent will women accept HPV self-sampling for cervical cancer screening? A qualitative study conducted in Switzerland. Int. J. Womens Health.

[21] Howard M., Lytwyn A., Lohfeld L., Redwood-Campbell L., Fowler N., Karwalajtys T. (2009). Barriers to acceptance of self-sampling for human papillomavirus across ethnolinguistic groups of women. Can. J. Public Health.

[22] Nishimura H., Yeh P.T., Oguntade H., Kennedy C.E., Narasimhan M. (2021). HPV self-sampling for cervical cancer screening: A systematic review of values and preferences. BMJ Glob. Health.

[23] Statista. https://www.statista.com/statistics/330695/number-of-smartphone-users-worldwide/.

[24] International Papillomavirus Society (IPVS). https://ipvsoc.org/news/fight-hpv-free-social-gaming-app/.

[25] Real F.J., Rosen B.L., Bishop J.M., McDonald S., DeBlasio D., Kreps G.L., Klein M., Kahn J.A. (2021). Usability Evaluation of the Novel Smartphone Application, HPV Vaccine: Same Way, Same Day, Among Pediatric Residents. Acad. Pediatr..

[26] Woodall W.G., Zimet G., Kong A., Buller D., Reither J., Chilton L., Myers V., Starling R. (2021). Vacteens.org: A Mobile Web app to Improve HPV Vaccine Uptake. Front. Digit. Health.

[27] Genomica. https://genomica-cp701.wordpresstemporal.com/wp-content/uploads/2021/12/CLART-Hpv4-INGLES.pdf.

[28] Solomon D., Davey D., Kurman R., Moriarty A., O’Connor D., Prey M., Raab S., Sherman M., Wilbur D., Wright T. (2002). Forum Group Members; Bethesda 2001 Workshop. The 2001 Bethesda System: Terminology for reporting results of cervical cytology. JAMA.

[29] Cohen J. (1960). A Coefficient of Agreement for Nominal Scales. Educ. Psychol. Meas..

[30] Bray F., Ferlay J., Soerjomataram I., Siegel R.L., Torre L.A., Jemal A. (2018). Global cancer statistics 2018: GLOBOCAN estimates of incidence and mortality worldwide for 36 cancers in 185 countries. CA A Cancer J. Clin..

[31] Koliopoulos G., Nyaga V.N., Santesso N., Bryant A., Martin-Hirsch P.P., Mustafa R.A., Schünemann H., Paraskevaidis E., Arbyn M. (2017). Cytology versus HPV testing for cervical cancer screening in the general population. Cochrane Database Syst. Rev..

[32] Chrysostomou A.C., Stylianou D.C., Constantinidou A., Kostrikis L.G. (2018). Cervical Cancer Screening Programs in Europe: The Transition Towards HPV Vaccination and Population-Based HPV Testing. Viruses.

[33] Gök M., Heideman D.A., van Kemenade F.J., de Vries A.L., Berkhof J., Rozendaal L., Beliën J.A., Overbeek L., Babović M., Snijders P.J. (2012). Offering self-sampling for human papillomavirus testing to non-attendees of the cervical screening programme: Characteristics of the responders. Eur. J. Cancer.

[34] Tranberg M., Bech B.H., Blaakær J., Jensen J.S., Svanholm H., Andersen B. (2018). Preventing cervical cancer using HPV self-sampling: Direct mailing of test-kits increases screening participation more than timely opt-in procedures—A randomized controlled trial. BMC Cancer.

[35] Haguenoer K., Sengchanh S., Gaudy-Graffin C., Boyard J., Fontenay R., Marret H., Goudeau A., Pigneaux de Laroche N., Rusch E., Giraudeau B. (2014). Vaginal self-sampling is a cost-effective way to increase participation in a cervical cancer screening programme: A randomised trial. Br. J. Cancer.

[36] Waller J., Bartoszek M., Marlow L., Wardle J. (2009). Barriers to cervical cancer screening attendance in England: A population-based survey. J. Med. Screen..

[37] Bos A.B., Rebolj M., Habbema J.D., van Ballegooijen M. (2006). Nonattendance is still the main limitation for the effectiveness of screening for cervical cancer in the Netherlands. Int. J. Cancer.

[38] Sultana F., Mullins R., English D.R., Simpson J.A., Drennan K.T., Heley S., Wrede C.D., Brotherton J.M., Saville M., Gertig D.M. (2015). Women’s experience with home-based self-sampling for human papillomavirus testing. BMC Cancer.

[39] Madzima T.R., Vahabi M., Lofters A. (2017). Emerging role of HPV self-sampling in cervical cancer screening for hard-to-reach women: Focused literature review. Can. Fam. Physician.

[40] Crofts V., Flahault E., Tebeu P.M., Untiet S., Fosso G.K., Boulvain M., Vassilakos P., Petignat P. (2015). Education efforts may contribute to wider acceptance of human papillomavirus self-sampling. Int. J. Womens Health.

[41] Sauvaget C., Fayette J.M., Muwonge R., Wesley R., Sankaranarayanan R. (2011). Accuracy of visual inspection with acetic acid for cervical cancer screening. Int. J. Gynaecol Obs..

[42] Cuzick J., Arbyn M., Sankaranarayanan R., Tsu V., Ronco G., Mayrand M.H., Dillner J., Meijer C.J. (2008). Overview of human papillomavirus-based and other novel options for cervical cancer screening in developed and developing countries. Vaccine.

[43] Arbyn M., Simon M., de Sanjosé S., Clarke M.A., Poljak M., Rezhake R., Berkhof J., Nyaga V., Gultekin M., Canfell K. (2022). Accuracy and effectiveness of HPV mRNA testing in cervical cancer screening: A systematic review and meta-analysis. Lancet Oncol..

[44] Tranberg M., Jensen J.S., Bech B.H., Blaakær J., Svanholm H., Andersen B. (2018). Good concordance of HPV detection between cervico-vaginal self-samples and general practitioner-collected samples using the Cobas 4800 HPV DNA test. BMC Infect. Dis..

[45] Cho H.W., Ouh Y.T., Hong J.H., Min K.J., So K.A., Kim T.J., Paik E.S., Lee J.W., Moon J.H., Lee J.K. (2019). Comparison of urine, self-collected vaginal swab, and cervical swab samples for detecting human papillomavirus (HPV) with Roche Cobas HPV, Anyplex II HPV, and RealTime HR-S HPV assay. J. Virol. Methods.

[46] Smith L.W., Khurshed F., van Niekerk D.J., Krajden M., Greene S.B., Hobbs S., Coldman A.J., Franco E.L., Ogilvie G.S. (2014). Women’s intentions to self-collect samples for human papillomavirus testing in an organized cervical cancer screening program. BMC Public Health.

[47] FUTURE II Study Group (2007). Quadrivalent vaccine against human papillomavirus to prevent high-grade cervical lesions. N. Engl. J. Med..

[48] Plummer M., Herrero R., Franceschi S., Meijer C.J., Snijders P., Bosch F.X., de Sanjosé S., Muñoz N. (2003). IARC Multi-centre Cervical Cancer Study Group. Smoking and cervical cancer: Pooled analysis of the IARC multi-centric case--control study. Cancer Causes Control.

